# Membrane Permeability Monitoring to Antipsychotic Olanzapine Using Platinum Black-Modified Electrodes

**DOI:** 10.3390/s25072266

**Published:** 2025-04-03

**Authors:** Murugaiya Sridar Ilango, Dayananda Desagani, Srikanth Jagadeesan, Alexander Snezhko, Gad Vatine, Hadar Ben-Yoav

**Affiliations:** 1 Nanobioelectronics Laboratory, Department of Biomedical Engineering, Ben-Gurion University of the Negev, Beer Sheva 8410501, Israel; niranjansridar@gmail.com (M.S.I.); dayanandabits@gmail.com (D.D.);; 2 Department of Physiology and Cell Biology, Faculty of Health Sciences, Ben-Gurion University of the Negev, Beer Sheva 8410501, Israelvatineg@bgu.ac.il

**Keywords:** olanzapine, platinum black, blood–brain barrier, electrochemical sensors, schizophrenia

## Abstract

The blood–brain barrier (BBB) is key to the regular functioning of the central nervous system. The dysfunction of the BBB has been described in various neurological disorders, including schizophrenia. Schizophrenia (SCZ) is a chronic psychiatric disorder described by hallucinations, delusions, and negative symptoms. The Olanzapine (OLZ) drug is an electroactive species, and its levels can be monitored using electrochemical sensors. The detection of OLZ was demonstrated previously by using electrochemical sensors, and this technique can be used to monitor the levels of OLZ in real time. The challenge is to identify the permeability of OLZ through the BBB, so a replica model was designed with the BBB based on a Transwell membrane seeded with endothelial cells. A microfabricated electrode consisting of a 3 mm Au disk was modified with platinum black; this enables higher selectivity of electrochemical signals from OLZ. The dose–response of OLZ was characterized in phosphate buffer saline solution (10 mM, pH 7.4) by adding 20–200 nM (in steps 20) of OLZ stock solution. The observed chronoamperometric electrochemical signals showed an increasing current at 0.45 V vs. Ag/AgCl with an increasing OLZ concentration. The controls for the experiments were performed in phosphate-buffered saline solution (10 mM, pH 7.4). The detection limit was calculated as 9.96 ± 7.35 × 10^−6^ nM from the calibration curve. The membrane permeability of the OLZ drug tested with five SCZ patients was monitored by studying the TEER measurements and permeability rate constant data.

## 1. Introduction

Olanzapine (2-Methyl-4-(4-methyl-1-piperazinyl)-10H-thieno[2,3-b][1,5]benzodiazepine) is an antipsychotic drug used for schizophrenia (SCZ) diseases such as alogia, anhedonia, avolition, and other psychotic diseases [1,2,3]. Olanzapine (OLZ) is a widely used second-generation antipsychotic drug for the treatment of schizophrenia and bipolar disorder due to its broad-spectrum efficacy and fewer side effects compared to first-generation antipsychotics [2,4,5,6]. Schizophrenia is a multifactorial disease that can be treated most effectively using drugs that interact with multiple neurotransmitter systems [3,6,7]. Olanzapine is one of these drugs and shows a high affinity for neurotransmitters such as dopamine D1, D2, D4, serotonin 5-HT2A, 5-HT2C, 5-HT3, α1-adrenergic histamine H1, and muscarinic receptors [8,9,10,11].

The pharmacological action of OLZ largely depends on its ability to cross the blood–brain barrier (BBB) efficiently and interact with dopamine and serotonin receptors within the central nervous system (CNS) [12]. However, predicting its BBB permeability and systemic responsiveness remains a significant challenge in drug development and therapeutic optimization [13,14]. The role of the BBB is to control the transportation of various metabolites, which consist of amino acids, lipids, peptides, nucleic acids, carbohydrates, vitamins, and minerals, along with drugs used for CNS-related disease treatment, between the blood and brain compartments. In this way, the BBB acts as both a safeguard and preserves the interstitial conditions of the CNS [15]. It remains unclear to what extent OLZ, particularly at dosages near the upper limit of its therapeutic range, influences the function of BBB cells. Such effects may have significant implications for the transport of OLZ across the BBB, its pharmacokinetics, and its overall impact on CNS function and clinical outcomes. Notably, OLZ concentrations comparable to those observed in the serum of patients receiving high doses have been shown to induce substantial changes in BBB permeability [16,17]. Psychostimulant drugs of abuse alter BBB function and increase permeability, likely contributing to their associated neurotoxicities. For instance, acute administration of 5 mM chlorpromazine increases the permeability of the BBB to ^59^Fe^3+^, mannitol, and inulin [18,19]. This observed effect suggests a breach in the BBB at a drug concentration comparable to that occasionally detected in human serum (3 mM, 1000 ng/mL). Detecting OLZ in Transwell systems seeded with cells is crucial for understanding its pharmacokinetics and pharmacodynamics at the cellular level [20]. Accurate measurement of OLZ concentrations in this in vitro model helps to study its cellular uptake, distribution, metabolism, and potential cytotoxic effects. This information is vital for optimizing dosing regimens and improving therapeutic outcomes. Wang et al. investigated the penetration of OLZ into mice brains [16]. The results indicated that the expression of p-glycoprotien in the BBB significantly limits the penetration of olanzapine into the central nervous system. Moreover, the OLZ concentration was analyzed using the HPLC technique, which is a very expensive tool and time-consuming analysis. Traditional methods to assess BBB permeability, such as in vivo models and in vitro cell-based assays, often involve labor-intensive and time-consuming protocols [21]. Among in vitro techniques, the measurement of transepithelial electrical resistance (TEER) has been a cornerstone for evaluating BBB integrity, yet it lacks the precision to quantify drug penetration rates [22,23,24]. Developing sensitive, cost-effective, and reproducible analytical platforms is essential to overcome these limitations. Numerous analytical techniques have been reported for the quantification of OLZ in pure as well as in dosage form. These include high-performance liquid chromatography (HPLC) [9,25,26,27], mass spectroscopy (MS) [26], gas chromatography (GC) [28], GC-MS and liquid chromatography coupled with MS [26,29], spectrophotometry [30], and electroanalytical techniques [31] and in combination with these techniques [32]. However, traditional chromatographic, spectroscopic, and analytical methods exhibit several limitations, including low sensitivity, high sample volumes, expensive equipment, complex sample pretreatment and dilution requirements, and time-intensive extraction and separation procedures. In contrast, electroanalytical methods offer notable advantages, such as rapid response, simplicity, cost-effectiveness, broad applicability in both qualitative and quantitative analyses, high accuracy, and the potential for miniaturization. These distinctive attributes have led to the widespread adoption of electrochemical methods for the analysis of environmental and pharmaceutical samples in recent years.

There are different materials used for olanzapine estimation by electrochemical techniques. Azab et al. used polyethylene glycol and silver nanoparticles to improve the sensitivity of the carbon paste electrode for electrochemical estimation of OLZ [33]. Ahmed et al. used the voltammetric technique to determine olanzapine in tablets and human urine samples with modified carbon paste electrodes by incorporating gold nanoparticles and glutamine in a micellar medium [34]. Platinum-black-modified electrodes, in particular, offer enhanced surface area and catalytic activity, making them highly suitable for detecting low-concentration analytes [35,36,37,38,39]. Previous studies have demonstrated the utility of such sensors in monitoring small-molecule drugs, but their application in antipsychotic permeability assessments, especially for OLZ, remains unexplored.

In our previous study, the OLZ was analyzed by an electrochemical technique using platinum-black-modified microelectrodes [35]. The OLZ content in undiluted serum with a limit of detection (LOD) of 28.6 ± 1.3 nM and a sensitivity of 0.14 ± 0.02 μA/cm^2^ nM was analyzed. In this study, platinum-black-modified electrodes were utilized to monitor OLZ concentrations across an in vitro BBB model. The sensor-based data were correlated with TEER measurements to provide insights into OLZ permeability dynamics. The present work on the OLZ sample diluted with organic solvent achieved an LOD of 9.96 ± 7.35 × 10**^−^**^6^ nM and a sensitivity of 0.027 ± 0.001 μA/cm^2^ nM. In this study, the permeability of OLZ through the BBB was investigated. The OLZ concentration was analyzed using an electrochemical technique using the platinum-black-modified electrode.

## 2. Materials and Methods

The following chemicals and materials are used in the experiment: OLZ (CAS number: 132539-06-1, Sigma-Aldrich, College park, MD, USA), sodium chloride (CAS number: 7647-14-5, Merck, Nantong, China), 2-propanol (CAS number: 67-63-0, Bio-Lab, Ltd., Jerusalem, Israel), potassium hexacyanoferrate (II) trihydrate (‘Ferrocyanide,’ CAS number: 14459-95-1, Merck, Darmstadt, Germany), Dihydrogen hexachloroplatinate (IV) hexahydrate (chloroplatinic acid; CAS number: 26023-84-7, Alfa Aesar, Petach Tikva, Israel), potassium hexacyanoferrate(III) (‘Ferricyanide,’ CAS number: 13746-66-2, Merck, Darmstadt, Germany), 99% Lead(II) acetate trihydrate (lead acetate; CAS number: 6080-56-4, Alfa Aesar, Petach Tikva, Israel), hydrochloric acid 32% (CAS number: 7647-01-0, Bio-Lab, Ltd, Jerusalem, Israel), di-sodium hydrogen phosphate dehydrate (CAS number: 10028-24-7, Merck, Raanana, Israel), sodium dihydrogen phosphate dihydrate (CAS number: 13472-35-0, Merck, Darmstadt, Germany), acetone (CAS number 67-64-1, Sigma-Aldrich, Darmstadt, Germany), and deuterium- depleted water (>18 MΩ) from Millipore (Millipore system, Thermo Scientific, Waltham, MA, USA). The solutions were diluted using PBS solution (10 mM, pH 7.4). The electrochemical experiments were conducted using the three-electrode system, a platinum wire counter electrode (catalog number: 012961, ALS Co., Ltd, Tokyo Japan), an Ag/AgCl reference electrode (catalog number: 011464, BAS, Inc., Kent Avenue, West Lafayette, USA), and an in-house fabricated gold electrodes on a glass substrate as the working electrode.

The OLZ solutions were prepared in 2-propanol [35] and stored at −20 °C. The calibration was performed by mixing the OLZ solution with PBS (10 mM, pH 7.4). The preparation of the platinum black solution [40], fabrication of the electrodes, and electrodeposition of the platinum black solution are elaborated on in the Supplementary File. The effective surface area of the platinum-black-modified electrodes was calculated using the Randles–Sevick relationship [41,42] is 5.23 × 10^−2^ ± 2.3 × 10^−3^ cm^2^, which is higher than the bare Au electrode, 3.81 × 10^−2^ ± 1.2 × 10^−3^ cm^2^. The higher surface area is due to non-identical and uneven roughness on the surface of the platinum black [43].

**Figure 1:**
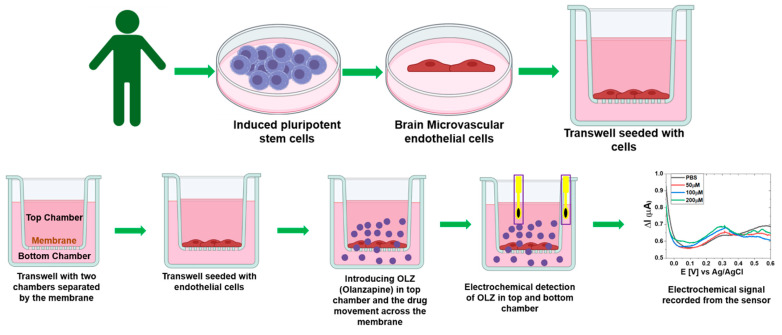
Schematic of preparing the BBB membrane from human induced pluripotent stem cells in a dual Transwell separated by a membrane and the introduction of OLZ with electrochemical measurements.

The induced pluripotent stem cells are collected from five different cell lines (Labelled as Line 1, 3, 5, 7, and 9) and used to prepare brain microvascular endothelial cells. These cells were transferred to the Transwell to identify the movement of OLZ through them to analyze the effect of the OLZ drug on different individual lines. The preparation of the Transwell is explained schematically in Figure 1. The samples were collected at five different times (0, 3, 6, 9, and 12 min) from the basolateral side. We collected a drug sample immediately after introducing it in the upper chamber to determine its initial concentration. Detecting the concentration of drugs that permeate from top to bottom was the objective. Hence, we collected and detected drug concentrations in the lower chamber. The electrochemical behavior of the OLZ was measured using chronopotentiometry before and after inducing the drug for all the individual lines at different concentrations with different potentials.

TEER measurements on Transwells were taken using an STX2 electrode and an EVOM2 Voltammeter (World Precision Instruments, Sarasota, FL, USA). This epithelial voltammeter uses two pairs of electrodes, one applying current and the other measuring the output voltage, on each side of a monolayer, for example, a Transwell membrane [23]. After 24 h of plating iBMECs onto Transwells, TEER measurements were taken every 24 h for three subsequence days. The STX2 electrode was positioned within the well and the resistance (Ω) was taken once equilibrated, before repeating in two more locations on each Transwell, to calculate the mean resistance. The resistance of the BBB is measured to identify the dose requirement for different individuals. The correlation between the TEER value and permeability was investigated to indicate the effect of OLZ on different individuals.

The platinum-black-modified electrodes were used to detect OLZ, and their electrochemical signals were recorded for the stock solution and solutions obtained from the Transwell. A chronoamperometry (CA) technique with a potential of 0.45 V was used to calibrate the response of OLZ. From the calibration curve, the LOD and sensitivity were analyzed. The response and the OLZ concentration curve were plotted to find the slope of the linear regression, which shows the sensitivity, and three standard deviations give the LOD. The modified electrodes were used to test different concentrations of OLZ ranging from 20 to 200 nM in steps of 20 nM with three modified electrodes. These three platinum-black-modified electrodes were used for measuring the OLZ signals with 10 repeats. The background signal was taken from 10 mM PBS solution with 40 repeats. After every measurement, the electrodes were cleaned with deionized water and dried using nitrogen gas.

## 3. Results and Discussion

### 3.1. Olanzapine Sensing

**Figure 2:**
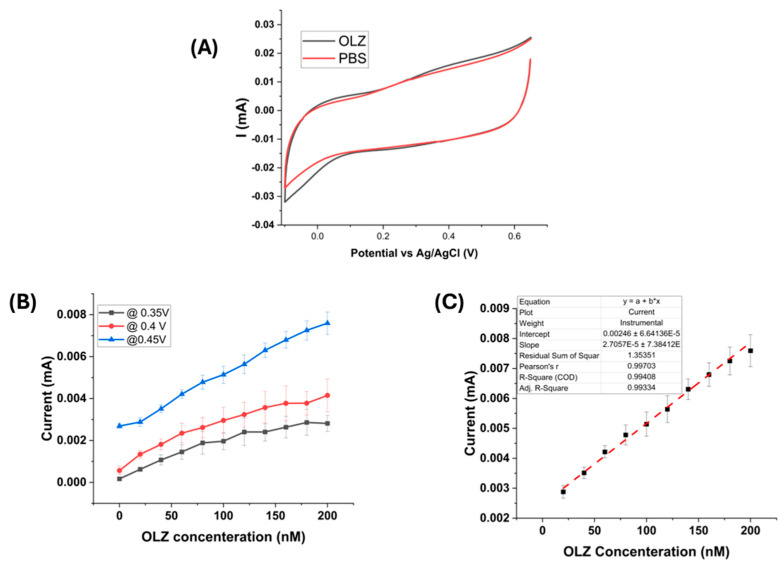
(**A**) Cyclic voltammetry of 100 nM OLZ in PBS solution and PBS solution, (**B**) Chronoamperometric current measured at 20 s for different concentrations of OLZ with three different potentials (0.35, 0.4, and 0.45 V), (**C**) Dose–response plot for platinum-black-modified electrode at 0.45 V.

The sensing performance of the platinum-black-modified electrode for olanzapine was characterized with cyclic voltammetry (CV) of 100 nM OLZ in PBS solution and compared with CV of PBS solution (Figure 2A). The plot showed the peak around 0.4 V. To further characterize the OLZ, the chronopotentiometry technique was used by measuring current at three different potentials 0.35, 0.4, and 0.45 (Figure 2B). The response at 0.45 V shows a linear correspondence with the increase in OLZ concentration from 20 nM to 200 nM. So, the response at 0.45 V is considered for further analysis and sensitivity, and LOD calculations (Figure 2C) from Equations (1) and (2).

LOD = (3 × Std. Dev of PBS)/Slope of the analyte from the calibration curve (1)

Error of LOD = (3 × Std. Dev × Error of slope)/(slope)^2^ (2)

The sensitivity of the measured response was 0.027 ± 0.001 µA/cm^2^, and LOD was 9.96 ± 7.35 × 10^−6^ nM.

### 3.2. Rate Constant for Precision Dosing of OLZ

The platinum-black-modified electrodes were used to test the response from the samples obtained (Transwell). The samples are obtained from schizophrenia patients. This approach to SCZ patients can contribute to understanding the molecular mechanism of SCZ and the pathophysiology of treatment resistance. Human-induced pluripotent stem cells (hiPSCs) give an interesting new road to investigate the function of BBB disruption [44]. We aimed to generate iPSCs from five SCZ patients. We utilized these iPSCs to induce pluripotent stem cell-derived brain microvascular endothelial cells (iBMECs) and developed in vitro models for the BBB to study the function in SCZ compared to HC. iBMECs were seeded on Transwell and barrier properties were evaluated by measuring TEER and paracellular permeability. The TEER values commonly examined the permeability of the epithelial and endothelial cell monolayers [45]. The values of TEER state the resistance to an electrical current passed through the cell monolayer to assess permeability to small inorganic ions [45]. Table 1 illustrates the variation in TEER values for different lines and also the effect of OLZ on the TEER value for all the membranes. Reduced TEER values indicate higher permeability and impaired barrier integrity. The permeability increased with the increase in OLZ concentration, which is the reason for the higher current observed in CA plots.

**Figure 3:**
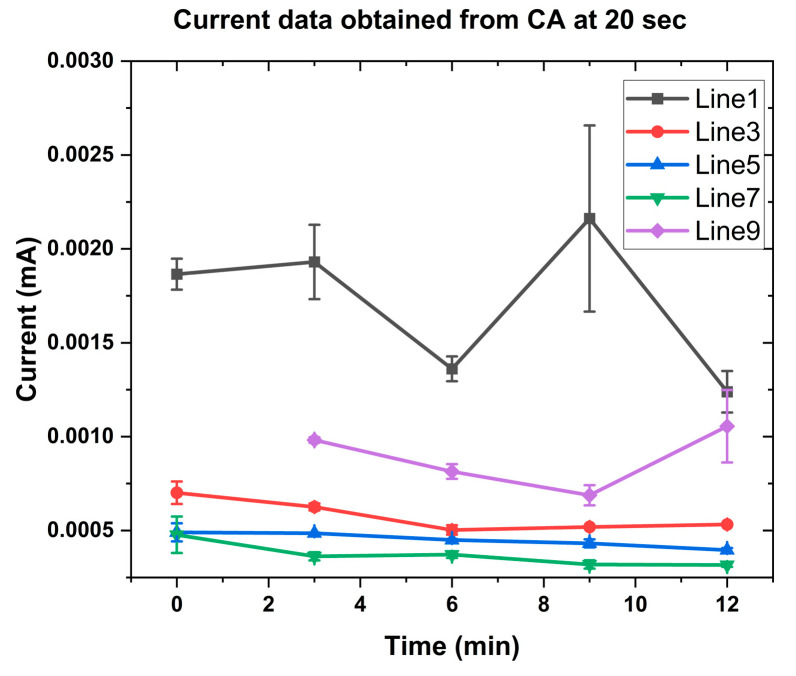
Current data from chronoamperometry measured at 20 s for different lines at 0.45 V.

The media were collected from five different patients from the bottom of the Transwell after adding OLZ drug at 100 nM concentration. The samples were collected at five different times (0, 3, 6, 9, and 12 min) from the time of OLZ addition. These samples were tested for OLZ response using platinum-black-modified electrodes for all the patients. Figure 3 shows the results of the measured current for different samples at 20 s of chronoamperometry (CA). Lower TEER values exhibit a high transfer of OLZ through the membrane, which is reflected in the higher current in CA. Line 1 shows a higher current, which has a lower TEER value compared to other cell lines [46].

**Figure 4:**
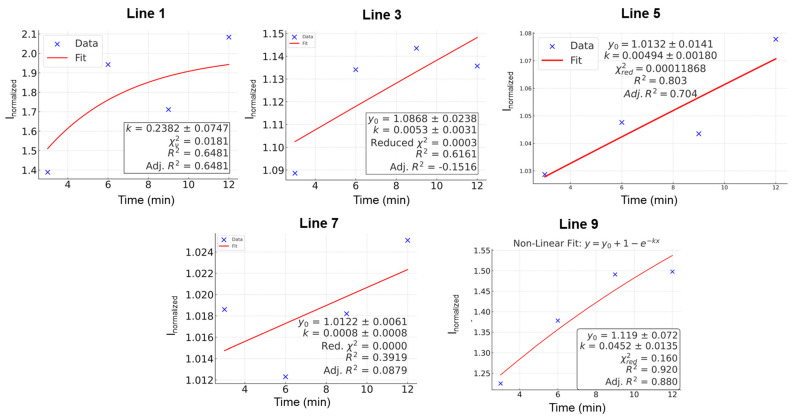
Permeability rate constant calculation of 5 SCZ patient’s cell lines (1, 3, 5, 7, and 9) from the normalized current data obtained after the drug transportation through the membrane.

The CA data obtained for all the cell lines at different times (0, 3, 6, 9, and 12 min) were normalized using the following equation. After normalization, the current at 20 s was used in Figure 4.

(3) $$I_{normalized}=\frac{I_{ij}}{\sqrt{\Sigma_{j}I_{ij}^{2}}}$$

where ‘**i**’ is the time [min] and ‘j’ represents current [A].

The permeability rate constant is calculated using the normalized response plot of the platinum-black-modified electrode (Equation (5)) [47,48].

(4) $$\mathrm{Normalized}\;\mathrm{concentration}=\frac{C_{t}}{C_{0}}=1-e^{-kt}$$

Here, $\frac{C}{C_{0}}\left(t\to \infty \right)=1\frac{C}{C_{0}}\left(t=0\right)=0$

(5) $$asC\left(t\right)\propto I\left(t\right),\;I_{\mathrm{normalized}}=1-e^{-kt}$$

where C**_t_** is the concentration of OLZ that passed the barrier (bottom area), C**_0_** is the initial concentration of OLZ that was in the top area, I is the response current [A], k is the rate constant, and t is the time [min].

**Figure 5:**
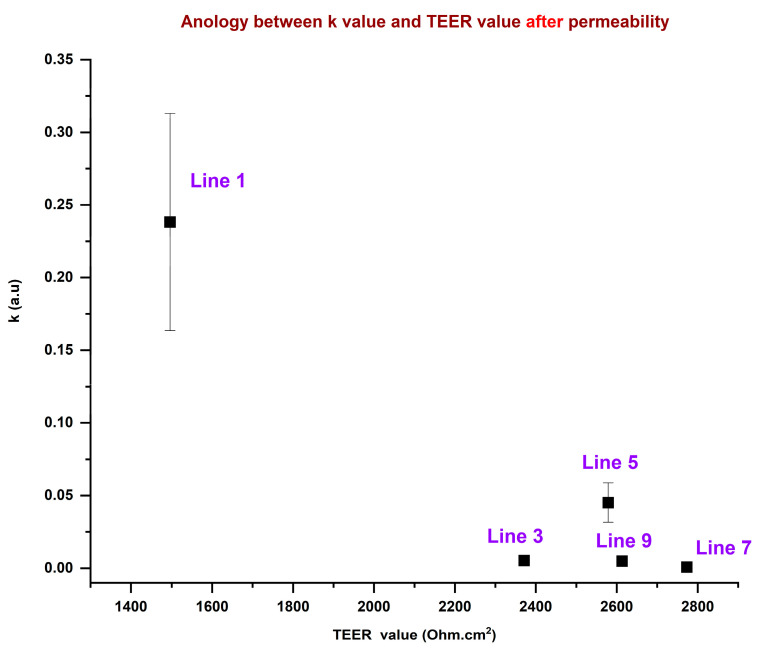
Correlation between the TEER value and permeability rate constant of OLZ.

The above equation can qualitatively describe drug transport through a membrane, particularly in scenarios where drug accumulation follows first-order kinetics. The equation assumes a single-compartment model and may not fully capture complex active transport mechanisms or saturation kinetics (e.g., facilitated diffusion or carrier-mediated transport). The rate constant describes how fast the drug permeates through the membrane. It is directly influenced by factors such as membrane thickness, drug lipophilicity, surface area available for diffusion, and drug concentration gradient [49,50]. If passive diffusion is the primary transport mechanism, this equation provides an exponential growth model for drug accumulation. The model is plotted with an orthogonal distance regression iteration algorithm to fit the normalized current data from each line. Figure 4 shows the fitting of normalized current data with Equation (5) to determine the permeability rate constant. The nonlinear fitting of the data (Figure 4) for all the cell lines is not the best fit; however, to calculate the permeability rate constant, we considered the drug accumulation as first-order kinetics and calculated the rate constant. The rate at which drug transport through the membrane approaches equilibrium is determined by the permeability rate constant. A higher k means faster transport. The k values determined from the nonlinear fitting (Figure 4) are plotted along with TEER values in Figure 5 for the five SCZ patients (cell lines 1, 3, 5, 7, and 9). The data show that SCZ patients of lines 1 and 5 have faster transportation of the OLZ drug through the membrane, whereas 3, 7, and 9 cell line patients have slower transportation of the OLZ drug. The TEER value is a measure of ionic permeability through intercellular clefts [45]. Estimation of TEER guarantees noninvasive determination, which can also evaluate the barrier integrity of epithelial or endothelial cells at various stages of differentiation and growth [23]. The TEER is influenced by other parameters, including medium composition, temperature, and use of the equipment [51]. These parameters may also cause changes in the TEER values. The correlation between the TEER values and the permeability rate constant for the transport of the OLZ drug across the membrane is illustrated in Figure 5. Based on the TEER data and the predicted permeability rate constants, the membrane permeability of OLZ across the cell lines derived from five SCZ patients follows the order: Line 7 < Line 9 < Line 3 < Line 5 < Line 1. However, it is important to note that the TEER values and permeability rate constants do not exhibit a consistent trend across all cell lines; moreover, they are from five different SCZ patients. This discrepancy can be attributed to differences in the experimental methods and instrumentation used for data collection.

For TEER measurement, the positioning of the chopstick electrodes is critical and requires careful attention, as improper placement can lead to inaccurate resistance readings when compared to measurements obtained through other techniques, such as electrochemical analysis using a potentiostat. In some cases, it may be difficult to accurately assess high drug permeability using TEER measurements, due to errors introduced by inaccurate resistivity values. Furthermore, the integrity of the cell monolayer may be compromised during transfer procedures or due to fluctuations in environmental conditions, such as temperature and pH, which can lead to significant variations in TEER measurements.

Additionally, the reproducibility of TEER measurements using chopstick electrodes presents a notable challenge, as variations in electrode positioning, the limited surface area of the electrodes, and their geometry relative to the membrane can all contribute to inconsistencies in the data [52]. These factors complicate the comparison of TEER values and permeability rate constants across different patients. In this study, we attempted to correlate TEER with the permeability rate constant, which was estimated by fitting current data to Equation (5); however, the fit was not optimal. This poor fitting further explains why TEER values and permeability rate constants did not exhibit a strong correlation in this analysis. Based on the current data, a decrease in the TEER value shows that the barrier has become less intact, which means higher permeability and the same trend was observed in Figure 5, where Line 1 has a higher rate constant and lower TEER value.

## 4. Conclusions

This study highlights the potential of electrochemical sensors for predicting human responsiveness to antipsychotic drugs. Platinum-black-modified electrodes were employed to detect the concentration of OLZ after their penetration through the BBB in various cell line models. The effective surface area of the platinum-black-modified electrodes is 5.23 × 10^−2^ ± 2.3 × 10^−3^ cm^2^, which is four times higher than the bare gold electrode, 3.81 × 10^−2^ ± 1.2 × 10^−3^ cm^2^. The dose–response of OLZ with platinum-black-modified electrodes was characterized using chronoamperometric electrochemical signals, which showed an increasing current at 0.45 V vs. Ag/AgCl with an increasing OLZ concentration. The detection limit of OLZ was calculated as 9.96 ± 7.35 × 10^−6^ nM. The OLZ concentration data were utilized to predict its permeability rate, which was further compared with the TEER values. Based on the TEER values and predicted permeability rate constant data, the decreasing order of the membrane permeability of the OLZ drug tested with five SCZ patients is Line 1, Line 5, Line 3, Line 9, and Line 7. Here, we were able to monitor the olanzapine permeability across the membrane, which demonstrates the efficacy of platinum-black-modified electrodes as a robust analytical tool for evaluating antipsychotic drug transport.

## Figures and Tables

**Table 1:** TEER value of each line taken before the permeability of OLZ and after the permeability of the OLZ.

Lines	Line 1 (Ω cm^2^)	Line 3 (Ω cm^2^)	Line 5 (Ω cm^2^)	Line 7 (Ω cm^2^)	Line 9 (Ω cm^2^)
Before Permeability	1879	2689	2877	3145	2851
After Permeability	1496	2371	2613	2773	2579

## Data Availability

The data used to develop all the figures are not available to share publicly.

## Supplementary Materials

The following supporting information can be downloaded at: https://www.mdpi.com/article/10.3390/s25072266/s1, Figure S1: Schematic representation of the fabrication of gold electrodes. Figure S2: (A) Electrodeposition of platinum black using chronopotentiometry; (B) CV measured in 5 mM ferrocyanide/ferricyanide at different scan rates (0.05 to 0.5 V/s) using bare gold electrode; (C) CV measured at different scan rates for the platinum-black-modified electrode; plot of oxidation peak current vs. square root of the scan rate of (D) bare gold and (E) platinum-black-modified electrode; and (F) Optical microscopic image of platinum-black-modified electrode. Reference [53] is cited in the Supplementary Materials.
