# Supplementary material for: Membrane Permeability Monitoring to Antipsychotic Olanzapine Using Platinum Black-Modified Electrodes
Source: Sensors (Basel). 2025 Apr 3;25(7):2266. doi: 10.3390/s25072266 (PMC11991649; doi:10.3390/s25072266)
Supplement: Supplementary file 1 [file sensors-25-02266-s001.zip › sensors-3410470-supplementary.pdf]

Supplementary Data

Membrane Permeability Monitoring to Antipsychotic Olanzapine Using Platinum Black Modified Electrodes

Murugaiya Sridar Ilango[1, †], Desagani Dayananda[1, †], Srikanth Jagadeesan[2], Alexander Snezhko[1], Gad Vatine[2] and Hadar Ben-Yoav[1,*]

[1]Nanobioelectronics Laboratory, Department of Biomedical Engineering, Ben-Gurion University of the Negev, Beer Sheva, Israel

[2] Department of Physiology and Cell Biology, Faculty of Health Sciences, Ben-Gurion University of the Negev, Beer Sheva, Israel

[*]email: benyoav@bgu.ac.il

[†]These authors contributed equally to this work

Fabrication of gold electrodes:

Borosilicate glass was used as a substrate to fabricate the electrodes. The glass substrate was cleaned using acetone, isopropanol, and deionized water, and then dried using nitrogen gas. The substrate was dehydrated and baked for 120 seconds on a hot plate and then spin-coated with photoresist (AZ5214E) at 5000 rpm for 30 seconds. After that, it was soft-baked at 110 °C for 90 seconds. The substrate was exposed in photolithography in hard contact mode at 7.6 mW/cm^2 for 10 seconds using a mask aligner (Karl Suss Mask Aligner MA6). After exposure, the wafer was kept on a hot plate at 120 °C for 60 seconds (post-exposure step). After that, it was developed using AZ 726 MIF for 2 minutes and 45 seconds. The wafer was rinsed in deionized water and dried using nitrogen gas. The substrate was loaded in an e-beam evaporation system for depositing Ti/Au (20/200 nm). The substrate was immersed in acetone for the lift-off process and the patterned electrodes were obtained and observed through a microscope. The fabricated electrodes were cleaned using isopropanol and deionized water to remove the photo-resist residues from the wafer. The sample was then spin-coated using photoresist (SU8-3005) for patterning the chambers for the working electrode area. SU-3005 was spin-coated at 3000 rpm for 30 seconds and pre-baked at 95 °C for 15 minutes. The substrate is then exposed to UV light using a mask aligner (MA6, SUSS MicroTec) in hard contact mode for 50 seconds. The wafer was kept on a hot plate for post-exposure bake at 95 °C for 5 minutes. The wafer was developed using a PGMA ERB developer for 8 minutes and immersed in IPA before rinsing in deionized water. The substrate was placed on a hot plate at 150 °C for 5 minutes for hard baking the SU-3005. Figure S1 shows the schematic representation of the fabrication steps. The wafer was diced using a dicing saw (ADT-

7100). The fabricated electrodes were cleaned using acetone, methanol, and isopropanol separately for 5 minutes in each solution.

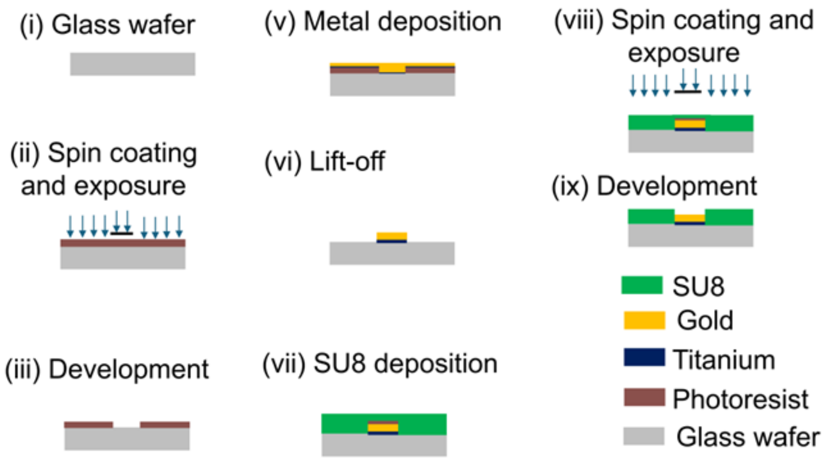

Figure S1: Schematic representation of the fabrication of gold electrodes.

The fabricated electrodes were cleaned using a piranha solution. The solution was prepared by mixing a 7:1 ratio of sulfuric acid and hydrogen peroxide. The electrodes were dipped in piranha solution for 25 seconds and rinsed with deionized water.

Preparation of Platinum black solution:

Briefly, 1% chloroplatinic acid and 0.05% lead acetate were mixed in deionized water to make a platinum black electro-deposition solution. Then, 0.0025% of 32% hydrochloric acid was added to the mixture and stirred [40]. The solution was stored in the dark and at room temperature.

Electrodeposition and Electrochemical characterization of the Platinum-black-modified electrode

The chronopotentiometry technique was used to electrodeposit platinum black on the gold surface. A current density of 23 mA/cm^2 was applied for 10 minutes to the electrodeposit platinum black on 3 mm disk Au electrodes. The modified electrodes were characterized a in 5 mM ferricyanide/ferrocyanide solution. Electrochemical activity of the platinum-black-modified

electrodes were tested using cyclic voltammetry (E_i=0V, E_1= -0.2 V, E_2 = 0.65 V, scan rate = 100 mV/s and repeat = 3).

Figure S2A shows the electrodeposition characteristics of the platinum black modification. The electrochemical signals demonstrated a higher oxidation and reduction current for the platinum-black-modified electrode than for the bare Au electrode because of the increase in the effective surface area. The effective surface area of the electrodes can be determined from the CV measurements at different scan rates of 0.05, 0.1, 0.2, 0.3, 0.4, and 0.5 V/s, using a 5 mM ferrocyanide/ferricyanide solution in the potential range of -0.2 to 0.65 V (Figure S2B,C). The values of the oxidation peak current for different scan rates were plotted against the square root of the scan rate, and the slope was calculated (Figure S2D, E).

Randles–Sevick relationship can be used to estimate the effective surface area of the platinum-black-modified electrodes, from equation S1 [53]:

$$I_p = 0.4463\ (\frac{F^3}{RT})^{1/2} n^{3/2} A_{eff} D^{1/2} C^* \nu^{1/2} \qquad \text{(S1)}$$

Here, I_p is the peak current [A], F is the Faraday constant [C/mol], T is the absolute temperature [K], R is the universal gas constant [J/mol K], n is the number of moles of electrons transferred in the cell reaction (n=1 for redox reaction ferrocyanide/ferricyanide), A_{eff} is the active surface area of the electrode [cm^2], D is the diffusion coefficient of the electroactive species [0.72* 10^{-5} cm^2/s for ferricyanide and 0.67* 10^{-5} cm^2/s for ferrocyanide [41]], C^* is the bulk concentration of the electroactive species [mol/cm^3], and ν is the linear potential scan rate [V/s].

By using equation S1, the value of A_{eff} is determined for the platinum-black-modified electrode, which is 5.23x10^{-2} ± 2.3x10^{-3} cm^2, and the effective surface area is higher than the bare Au electrode, which is 3.81x10^{-2} ± 1.2x10^{-3} cm^2. Wayu et al. investigated and reported that the increase in the surface area of the platinum-black-modified electrode is because of the non-identical and uneven roughness on the surface of the platinum black [43]. An optical microscopic image of the platinum-black-modified electrode is shown in Figure S2F.

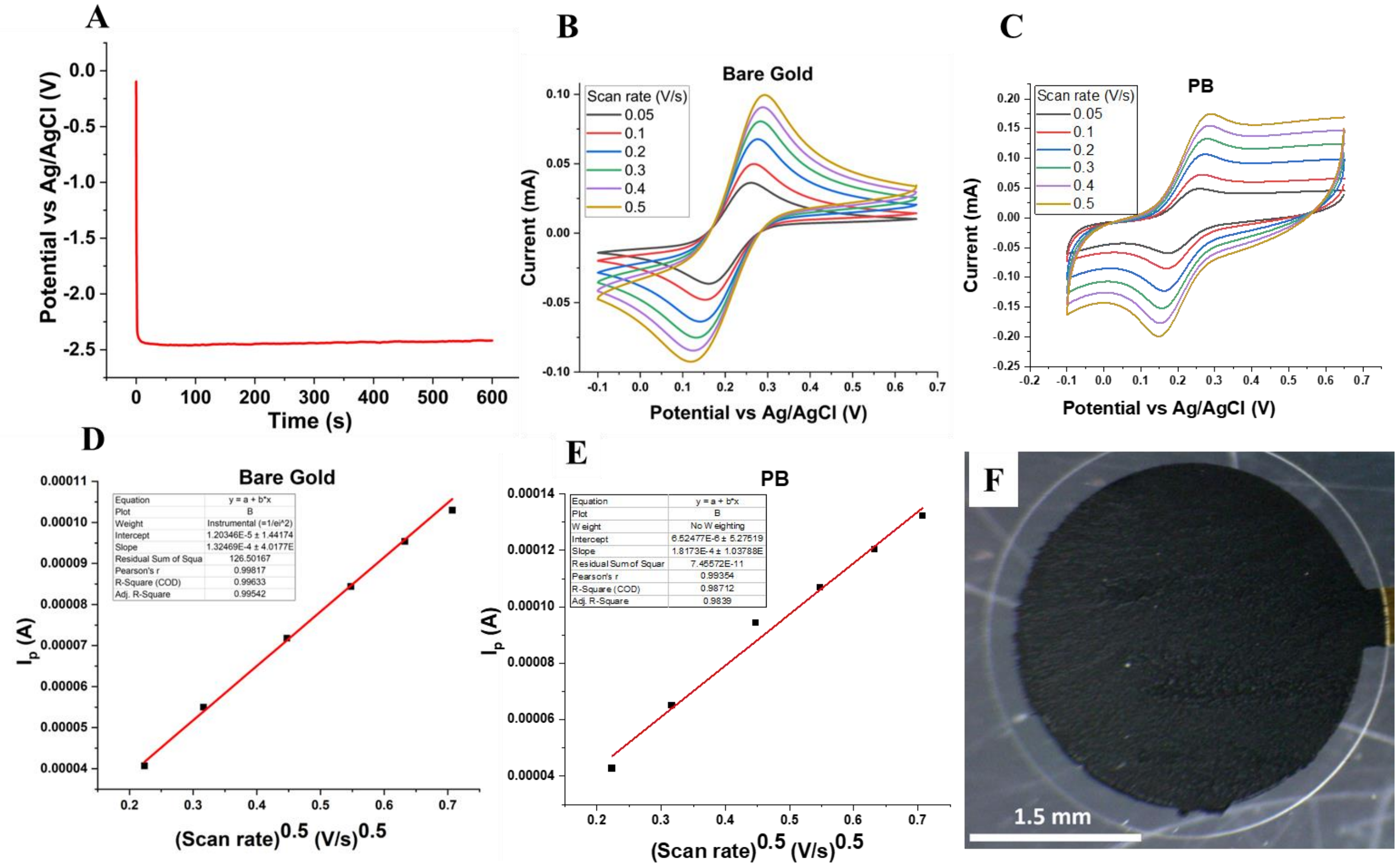

Figure S2: (A) Electrodeposition of platinum black using chronopotentiometry; (B) CV measured in 5 mM ferrocyanide/ferricyanide at different scan rates (0.05 to 0.5 V/s) using the bare gold electrode; (C) CV measured at different scan rates for the platinum black modified electrode; plot of oxidation peak current vs. square root of the scan rate of (D) bare gold and (E) platinum-black-modified electrode; and (F) Optical microscopic image of the platinum-black-modified electrode.
